# Review of the Palaearctic members of the *Lispe tentaculata* species-group (Diptera, Muscidae): revised key, synonymy and notes on ecology

**DOI:** 10.3897/zookeys.84.819

**Published:** 2011-03-01

**Authors:** Nikita Vikhrev

**Affiliations:** Zoological Museum of Moscow University, Bolshaya Nikitskaya 6, Moscow, 125009, Russia (ZMMU)

**Keywords:** *Lispe tentaculata*, *Lispe consanguinea*, *Lispe draperi*, *Lispe sericipalpis*, *Lispe orientalis*, *Lispe quaerens*, Muscidae, Diptera, key, Palaearctica, new synonym

## Abstract

The taxonomic reasons for regarding Lispe draperi Séguy, 1933, **sp. rev.**, as a valid species instead of a synonym of Lispe tentaculata (De Geer, 1776) and for treating Lispe quaerens Villeneuve, 1936, **syn. n.**, as a junior synonym of Lispe sericipalpis Stein, 1904 are given. A revised key for the Palaearctic members of the Lispe tentaculata species-group is given. Data on ecology, distribution and feeding preferences are provided.

## Introduction

The Lispe tentaculata species-group was proposed by Hennig (1960) and is characterized by the meron setulose above hind coxa and the following leg chaetotaxy: *t1* without setae; *t2* only with *p***-**seta, without *ad* or *av*, t3 with 1*ad* and 1 weak *pd*, without *av*.

Currently eight species are placed in the Lispe tentaculata species-group, six of which are present in the Palaearctic region. The taxonomic status of Lispe alpinicola Zhong, Wu & Fan, 1981 has not yet been settled (see below). Lispe sericipalpis Stein, 1904 and Lispe orientalis Wiedemann, 1824 are also distributed in the Oriental region. Lispe tentaculata (De Geer, 1776) is recorded from the very north of the Oriental region and widespread in Nearctic. Two more species of this group are found in the Nearctic region.

## Material and methods

The majority of the specimens studied are in the Zoological Museum of Moscow University (not indicated in text). Other material is in Natural History Museum, London (BMNH), Zoological Institute, St Petersburg (ZIN), Zoölogisch Museum, Universiteit van Amsterdam (ZMAN).

The following abbreviations for morphological structures are used: *f1*, *t1*, *f2*, *t2*, *f3*, *t3* = fore-, mid-, hind- femur or tibia; *ac* = acrostichal setae; *dc* = dorsocentral setae; *a*, *p*, *d*, *v* = anterior, posterior, dorsal, ventral seta(e); *prst* – presutural, *post* - postsutural.

Here I suggest a new abbreviation for the tarsi as *tar* followed by a pair of digits separated by a hyphen: the first digit (1 to 3) gives the leg number and the second digit (1 to 5) the number of the tarsal segment. For example, *tar1-4* = 4-th segment of fore tarsus; *tar3-1* = hind basitarsus.

## Identification key for Palaearctic species of the Lispe tentaculata species-group

**Table:** 

1	Males	2
–	Females	6
2	Fore tarsus modified: *tar1-1* yellow to dark, *tar1-2* to *tar1-4* yellow, *tar1-5* black; *tar1-1* half as long as *tar1-2*, with a dense row of brush-like *av* setulae and on posterior side with a finger-like yellow process with black apex, this process reaching middle of *tar1-2*; *tar1-2* projecting ventrally. Male cercal plate as Fig. 1	3
–	Fore tarsus unmodified. Male cercal plate as Fig. 3	5
3	Median third of *f3* with 2–5 *av* setae 1.5–2 times longer than femoral width. *tar3-1* shortened (slightly more than one third as long as *t3*); ventral median part of *tar3-1* concave; basal 1/3 of *tar3-1* with a brash of ventral hairs contrasting with shorter hairs in apical 2/3. Scutellum with some fine hairs below at apex (these hairs may be invisible in old specimens). Cercal plate with 2 pairs of projections apically, distinctly longer than wide (Fig. 1.2). 3 strong *post dc* setae	4
–	Median 1/3 of *f3* without long *av* setae (though in basal 1/3 of *f3* with 3–4 *av* subequal to femoral width). *tar3-1* straight, not curved or concave, *tar3-1* longer (slightly less than half as long as *t3*); ventral hairs on *tar3-1* of uniform length. Scutellum bare below at apex and bare at apex laterally below apical scutellar bristles. Cercal plate with only 1 pair of projections apically, almost as wide as long (Fig. 1.1). Usually 4 *post dc* setae, anterior 2 *post* pairs short and fine (but sometimes 3 strong *post dc* as in *tentaculata*!). Palearctic, temperate zone	Lispe consanguinea Loew
4	Male sternite 5 as in Fig. 2.1. Tibiae dark, yellow only at basal fifth. Median 1/3 of *f3* with 3–5 *av* setae. Basal 1/3 of *f3* with 3–4 *av* setae subequal to femoral width. Holarctic	Lispe tentaculata (De Geer)
–	Male sternite 5 as in Fig. 2.2. At least *t2* yellow in ground colour, more or less grey dusted, usually both *t2* and *t3* entirely yellow. Median 1/3 of *f3* with 2–3 *av* setae. Basal 1/3 of *f3* with 0–2 *av* setae subequal to femoral width. North Africa	Lispe draperi Séguy
5	Palpi black. *f2* with 2–7 fine *pv* setae in basal 1/3. *f3* with a long *v-pv* seta at base, 1–4 *av* setae in apical 2/5 and at most with a sparse row of 7–8 *pv*. *tar1-2* and *tar1-3* ventrally dark like the rest of fore tarsus. Presutural *ac* in 4–5 irregular rows. Dusting greyish. Length 5–6mm. Male terminalia – Figs 3.3, 4. Southern Palearctic and Oriental	Lispe sericipalpis Stein
–	Palpi yellow to brownish. *f2* with a full and dense row of 20–30 *pv* setae and full row of about 20 *av* (in both rows, setae long in basal half and short in apical half). *f3* with full rows of about 15 *av* and *pv* setae, the longest setae beyond middle twice as long as femoral width (long *v-pv* seta at base of *f3* also present, but not as conspicuous among other setae). *tar1-2* and *tar1-3* yellow ventrally. Presutural *ac* in 6–7 irregular rows. Dusting brownish. Length 6.5–7.5mm. Male terminalia – Figs 3.1, 2. Southern Palearctic and Oriental	Lispe orientalis Wiedemann
6	Only posterior pair of *prst dc* present. Presutural *ac* hairs weak and short, arranged in 5–7 irregular rows	7
–	Both pairs of *prst dc* present. Presutural *ac* hairs stronger, arranged in 3–4 rows	8
7	Palpi black (blackish brown in specimens collected 50–100 years ago). Presutural *ac* hairs in 5 rows. *f3* without av setae in basal 3/5. Length 5–6mm	Lispe sericipalpis Stein
–	Palpi yellow to light brown. Presutural *ac* hairs in 6–7 rows. *f3* with a row of 7–9 *av* in basal 3/5. Length 6–7mm	Lispe orientalis Wiedemann
8	Scutum with a median pruinose patch between 2-nd and 3-rd *post dc* setae; 4 strong *post dc* setae, 2nd and 3rd *post dc* closely approximated. (Scutellum with some fine hairs below at apex)	9
–	Scutum without median pruinose patch between 2-nd and 3-rd *post dc* setae; 3 strong *post dc* setae or only 2 strong *post dc* (and 1–2 weak anterior *post dc*), closely approximated strong post dc absent	10
9	Tibiae dark, only knees yellow. *f3* usually with 2–3 long submedian *av*; basal 1/3 of *f3* with 1–4 *av* subequal to *f3* width	Lispe tentaculata (De Geer)
–	*t2* at least in basal half yellow in ground colour, more or less dusted, usually both *t2* and *t3* entirely yellow. *f3* usually with only 1 long submedian *av*; basal 1/3 of *f3* with 0–3 *av* hardly longer than half *f3* width	Lispe draperi Séguy
10	Scutellum bare below at apex. *t2* and t3 yellow, more or less dusted. 2+4 *dc*, but the anterior 2 *post* pairs short and fine. *f3* in median 1/3 without *av* 1.5–2 times longer than femoral width (but 1–3 *av* setae subequal to *f3* width present at basal 1/3 of *f3*)	Lispe consanguinea Loew
–	Scutellum with some fine hairs below at apex (these hairs often invisible on old specimens). 2+3 *dc*, all strong. *f3* in median 1/3 with 1–3 *av* 1.5–2 times longer than *f3* width (females with male chaetotaxy of scutum)	Lispe tentaculata (De Geer)

**Figure 1.**
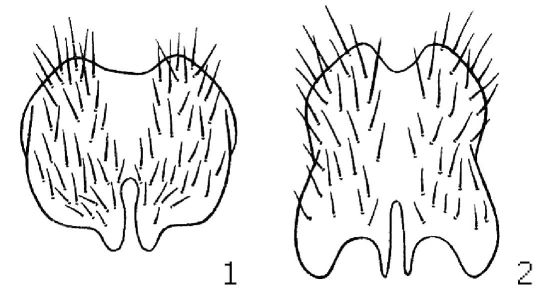
Male cercal plates. **1** Lispe consanguinea Loew **2** Lispe tentaculata (De Geer) (from Hennig, 1960).

**Figure 2.**
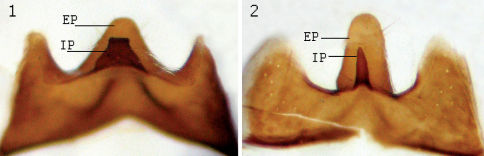
Male sternite 5, view from inner side. **1** Lispe tentaculata (De Geer) **2** Lispe draperi Séguy; **EP** external median process **IP** internal median process.

**Figure 3.**
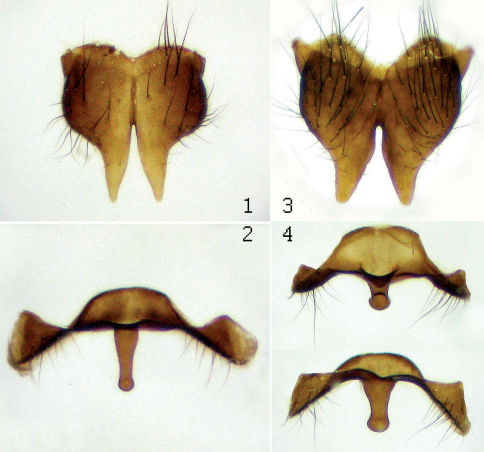
Male terminalia. **1, 2** Lispe sericipalpis Stein, **1** cercal plate **2** sternite 5; **3, 4** Lispe orientalis Wiedemann, **3** cercal plate **4** sternite 5 (from two points of view). <br/> In Lispe sericipalpis the cercal plate is more pointed, sternite 5 has the median process thinner and longer.

## Taxonomic notes

### Lispe alpinicola

Zhong, Wu & Fan, 1981

#### Remarks.

Described from Lhasa (China, 3500–4000 m asl.). Male fore and hind basitarsus modified as in Lispe tentaculata, female scutum with a pruinose patch as in Lispe tentaculata (Xue & Dong, 2005), male cercal plate similar to Lispe tentaculata (Xue & Chao, 1998). According to Xue and Dong (2005), males of Lispe alpinicola may be separated by the presence of a *p*-seta on *t1* and 3 *pd* setae on *t2*. It is difficult to give any opinion on this species without making a personal examination of specimens, but I have some doubts about the validity of this species. Among the rich *tentaculata* material examined, there were no specimens with both *p*-seta on *t1* and 3 *pd* setae on *t2*, but separately the presence of these setae was recorded. Several specimens from the north of Russia have a *p*-seta on *t1*. The male from Mongolia (about 2000 m asl.) has 2 *pd* setae on each mid tibia, and what is more these setae are placed differently on the left and right tibiae. See also remarks on Lispe tentaculata. For these reasons I have not included Lispe alpinicola in the identification key.

### Lispe consanguinea

Loew, 1858

#### Material examined.

Over 130 specimens. **Moldova;** **Russia (European):** Arkhangelsk reg., Chuvashia, Krasnodar reg., Moscow reg., Tver reg., Nizhnyi Novgorod reg., Vologda reg., Ulyanovsk reg.; **Mongolia; Russia (Asian):** Amur reg., Khanty-Mansi reg., Krasnoyarsk reg., Kurgan reg., Novosibirsk reg., Primorsky Kray; **Tajikistan:** Dushanbe; **Turkey:** Sakarya prov., Zonguldak prov.

### Lispe draperi

Séguy, 1933 sp. rev.

Lispa draperi Séguy 1933: 122. Fig. 11–13.
Lispe tentaculata ssp. draperi Canzoneri and Meneghini 1966: 115.

#### Material examined.

**Morocco:** east of Marrakech, 1400m, stones on river bank, 22.III.2009, N.Vikhrev, 1♀; west of Marrakech, Oued Nfiss, stones on river bank, 23.III.2009, N.Vikhrev, 2♂♂, 5♀♀; near Essaouira, stones on river bank and pond/pool silt, 24–29.III.2009, N.Vikhrev, 11♂♂, 4♀♀.

#### Remarks.

The conspecifity of the material listed above with type of Lispe draperi Séguy was kindly confirmed by A.C. Pont (pers. comm.) who examined the holotype of Lispe draperi in the Muséum national d’Histoire naturelle, Paris. Hennig (1960: 430) examined the type of this species and provisionally maintained it as a good species although he considered that the type might be an aberrant specimen of Lispe tentaculata. Canzoneri and Meneghini (1966) suggested that Lispe draperi is North African yellowish-legged subspecies of Lispe tentaculata but the species was sunk as a synonym of Lispe tentaculata by Pont (1986). This decision could be supported by the fact that the male cercal plate is similar to that of Lispe tentaculata. However, more careful examination shows that the structure of male terminalia differs and it is especially obvious in the structure of sternite 5 (Fig. 2). Lispe draperi should therefore be restored as a valid species, although closely related to Lispe tentaculata. I examined the sternite 5 of Lispe tentaculata collected in the Netherlands, Moscow region, Krasnodarsky Kray, Primorsky Kray (Russian Far East) and high in the Pamir mountains (Tajikistan, Gorno-Badakhshan, 3800 m asl.) and found it to be identical in all cases but different from that in Moroccan Lispe draperi.

### Lispe orientalis

Wiedemann, 1824

#### Material examined.

**Azerbaijan:** Lenkoran env., 38.66°N 48.79°E, 22–25.X.2008, N.Vikhrev, 16♂♂, 6♀♀.

**India:** Rajastan, Jaipur, 21.II. 2011, N.Vikhrev, 1♂, 1♀.

**Russia:** Krasnodarsky Kray, Sochi env., 43.547°N 39.811°E, 29.IX-24.X.2010, D.Gavryushin, 4♂♂; Primorsky Kray, 42.86°N 133.62°E, 18.IX.1987, A.Ozerov, 1♂.

**Tajikistan:** Khatlon div., Farkhor (=Parkhar) env., 37.420°N 69.352°E, 07.VI.2010, K.Tomkovich, 7♂♂, 5♀♀; Khatlon div., Kulob, 37.909°N 69.784°E, 07.VI.2010, K.Tomkovich, 19♂♂, 22♀♀.

**Thailand:** Mae Hong Son prov., 19.57N 98.28E, 650m asl., 21.XI.2010, N.Vikhrev, 1♂.

**Turkey:** Izmir prov., Dilek Milli Park, 37.68°N 27.10°E, 20.XII.2006, N.Vikhrev, 1♀; Antalia prov., Silion ruins, 36.989°N 30.985°E, goat drinking bowl, 25.V.2008, N.Vikhrev, 1♂, 1♀; Hatay prov., Arzus env., 36.407°N 35.886°E, 14.IV.2010, N.Vikhrev, 8♂♂, 2♀♀; Hatay prov., Samandag env., Çivlek, 17.IV.2010, N.Vikhrev, 6♂♂.

**S. Korea:** Seoul env., 31.VII.1938, Zhenzhurist, 5♀♀.

### Lispe sericipalpis

Stein, 1904

Fig. 4

Lispe sericipalpis Stein 1904: 110
Lispe quaerens Villeneuve 1936: 157, syn. n.

#### Material examined.

**Figure 4.**
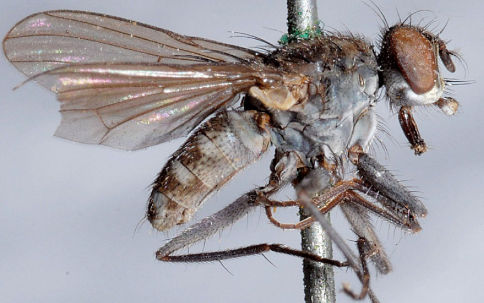
Lispe sericipalpis Stein, male lectotype (designated by Pont, 1970). Photo by Joke van Erkelens.

Lectotype male of *sericipalpis* – male (Fig. 4), paralectotypes 1♂, 4♀♀ (ZMAN).

**Azerbaijan:** Lenkoran reg., 38.65°N 48.80°E, 25.V.2009, K.Tomkovich, 1♀.

**Myanmar:** Shan state, Inle Lake env., 20.664°N 96.966°E, 26–30.XI.2009, N.Vikhrev, 4♂♂, 2♀♀; Kakaw env., 20.64°N 96.59°E, 03.XII.2009, N.Vikhrev, 45♂♂, 3♀♀.

**Nepal:** Solukhumbu distr., Janbesi env., 27.581°N 86.548°E, 2660m asl., 19.III.2010, A.Reshchikov, 2♂♂, 2♀♀.

**Tajikistan:** Dushanbe division, Ramit env. (38.72N 69.32E), river bank, 15–16.VI.2010, K.Tomkovich, 17♂♂, 30♀♀; Dushanbe env., 13.V.1943, A.Stackelberg, 1♂ (with handwritten label by W.Hennig “Lispe quaerens”) (ZIN); Varzob Canyon, 28–29.VII.1939, L.Zimin, 4♀♀ (ZIN); Varzob Canyon, 04.VII.1937, A.Gussakovsky, 1♂ (ZIN).

**Turkey:** Antalia prov., Köprü River, 37.075°N 31.232°E, 06–10.IX.2009, N.Vikhrev, 40♂♂, 29♀♀ (3♂♂, 2♀♀ deposited in BMNH); Mersin prov., 37.194°N 34.810°E, forest stream, 23.IV.2010, N.Vikhrev, 1♀; Bolu prov., 40.498°N 31.890°E, forest stream, 1800m asl., 31.VIII.2009, N.Vikhrev, 1♂; Sarakya prov., Karasu env., 41.03°N 30.79°E, forest stream, 15.VI.2010, N.Vikhrev, 1♀, 28.VIII.2009, N.Vikhrev, 1♂, 1♀; Zonguldak prov., Alaply env., 41.14°N 31.36°E, forest stream, 21.VI.2010, N.Vikhrev, 3♂♂, 2♀♀.

#### Remarks.

Male. Ground colour black. Pollinosity grey, but may be yellowish-grey. Palpus black(ish), but becoming brown in old specimens. Fronto-orbital plate and parafacial whitish, rarely yellowish. Scutum with 3 brown vittae along *ac* and *dc* rows, submedian (*dc*) vittae sometimes almost indistinct. *dc* 2(1) + 4 (rarely 3), as: presutural: weak to hardly distinct, medium; postsutural: weak, weak, strong, strong. Presutural *ac* in 4–5 irregular rows. Legs dark. *f2* with several (2–7) fine *pv*-setae in basal 1/3, the longest one (1.5 times as long as femoral width) at base. *f3* at base with long (1.5–2 times as long as femoral width) and fine characteristic seta in *v-pv* position. Other setae on *f3* variable: several *av*-setae present in apical third or slightly more, 1–3 among them longer, from as long as femoral width to twice as long; *pv* row may consist of 7–8 setae of which 2–3 are as long as femoral width, or may be reduced to 1–2 hardly distinct *pv* at base; sometimes chaetotaxy of *f3* differs on right and left legs of the same specimen. Abdomen whitish-grey to yellowish-grey dusted, with paired L-shaped, more or less extensive dark spots on tergites 3 to 5, divided by dusted median vitta. Female differs from male as follows: parafacial more often yellowish; *dc* 1+4(3), anterior *prst dc* always absent; *v-pv* seta at base of *f3* shorter to reduced; other ventral setae on *f2* and *f3* shorter or reduced.

The type locality of Lispe sericipalpis is Indonesia, Java. The species has also been recorded from other Indonesian islands (Bali, Sumatra), Taiwan, Vietnam, Thailand, Myanmar, Sri Lanka, India, Pakistan, Nepal. The type locality of Lispe quaerens is Turkey, Akshehir prov. This species, as interpreted by Hennig (1960: 453) who studied the holotype, has also been recorded from Spain, Italy, Croatia, several provinces of Turkey, Azerbaijan, Tajikistan and China. Thus, there appears to be no geographic gap between the natural habitats of Lispe sericipalpis and Lispe quaerens, but the former, known as an Oriental species, has never been compared with the latter, regarded as a Palearctic species. The examined series from Turkey and Tajikistan (some specimens were identified by Hennig, 1960: 453) had been assigned to Lispe quaerens, whereas the series from the Oriental region (Myanmar) had been assigned to Lispe sericipalpis. I came to conclusion that all the material listed above (Azerbaijan, Myanmar, Nepal, Tajikistan and Turkey) is conspecific with type series of Lispe sericipalpis. The terminalia of males from Turkey, Tajikistan, Nepal and Myanmar are similar. Oriental specimens have the dark abdominal patterns more extensive, especially so on tergite 3. Stein’s type series has the *av* setae on *f3* longer and more numerous in males, but all these characters may be found in some Palearctic specimens too.

Hennig (1960: 409) mentioned the possibility that Lispe quaerens could be a subspecies of Lispe orientalis. I am sure it is not - Lispe sericipalpis (= Lispe quaerens) is closely related to Lispe orientalis, but the two can be reliably separated, even as females (see key). In addition to morphological characters, there is a clear difference in ecology between these species (see below).

### Lispe tentaculata

(De Geer, 1776)

#### Material examined.

Over 350 specimens from a vast territory from the Iberian to Kamchatka Peninsulas.

**Europe:** **Greece;** **Latvia;** **Portugal;** **Russia (European):** Arkhangelsk, Chuvashia, Komi, Krasnodar, Kursk, Moscow, Murmansk, N. Ossetia, Nizhnyi Novgorod, Tula, Vladimir, Ulyanovsk; **the Netherlands;** **Ukraine.**

**Asia:** **Abkhazia; Armenia; Azerbaijan; Kazakhstan:** Almaty; **Mongolia:** Uvs prov.; **Russia (Asian):** Altai, Amur reg., Kamchatka, Khanty-Mansi reg., Krasnoyarsk reg., Magadan reg., Omsk reg., Primorsky Kray**,** Sakha (Yakutia) reg., Tyumen reg., Yamalo-Nenets reg.; **Tajikistan:** Dushanbe div., Khatlon div., Gorno-Badakhshan div.; **Turkmenistan:** Ahal, Mary; **Turkey (Asian):** Adana prov., Ankara prov., Antalya prov., Bolu prov., Duzce prov., Hatay prov., Isparta prov., Izmir prov., Kayseri prov., Konya prov., Mersin prov., Sakarya prov., Zonguldak prov.; **Uzbekistan:** Tashkent.

#### Remarks.

Lispe tentaculata has a variable *t3* chaetotaxy: besides the normal strong *ad* and short *pd* setae, an additional seta just below the strong *ad* but in a more *a*-position is often present and sometimes a second strong *ad* and a short *pd* may also be present. About 5% of females have a male-like scutum: with only 3 strong *post dc* and without a pruinose patch on the scutum.

## Distribution and ecology

Lispe tentaculata is a very common species across its natural habitat. The northernmost specimens were collected near Murmansk (forest-tundra at 69°N) and Vorkuta (tundra at 67.5°N), where this species was found on boulders on river banks and was the only Lispe species recorded at these places. In southern Turkey at 36°N Lispe tentaculata was common again on boulders in streams and, in springtime, at various muddy places such as temporary pools. In temperate conditions of the East European Plain Lispe tentaculata is absent from small forest streams and is infrequent on the sandy banks of big rivers but is common on the mud ponds and small lakes. Both in temperate and southern habitats Lispe tentaculata distinctly avoids a salty mud where it is replaced by species from the Lispe caesia-group and the Lispe palposa-group. Lispe tentaculata is common in high mountain areas where it prefers boulders along the shores of mountain lakes; the series from Gorno-Badakhshan in Tajikistan was collected at 3800 m asl.

Lispe draperi was observed in Morocco in late March and seems to have an ecology similar to Lispe tentaculata.

Lispe consanguinea clearly prefers narrow sandy bands along the banks of big rivers. In this habitat Lispe consanguinea is the dominant species in the temperate zone, whereas Lispe tentaculata is uncommon. I had never collected Lispe consanguinea in localities north of the Arctic Circle, but on sandy banks of the Vychegda River at 61.3°N 46.9°E this species was very common. The most southern records are Tajikistan (Dushanbe env., 1 specimen) and N. Turkey (2 specimens among numerous Lispe tentaculata).

Lispe sericipalpis (Fig. 5.1) may be found on boulders on the banks of rapid streams together with Lispe tentaculata. In N. Turkey this species is uncommon, whilst in S. Turkey Lispe sericipalpis is absent in spring time (late April), but becomes about as common as Lispe tentaculata in September. In Myanmar Lispe sericipalpis was absent in tree-shaded parts of streams, but was found in sunny sites at altitudes of 1100–1300 m asl.

**Figure 5.**
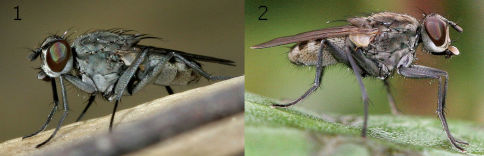
**1** Lispe sericipalpis Stein, male (Turkey, Antalya) **2** Lispe orientalis Wiedemann, male (Azerbaijan).

The ecology of Lispe orientalis (Fig. 5.2) is rather unusual: it is a species of dirty, organically-polluted water. Lispe orientalis was collected in Krasnodarsky Kray (Russia) and Antalya prov. (Turkey) around drinking bowls at pools polluted by cattle dung, in Tajikistan (Farkhor) at a very dirty irrigation ditch inside the town and at pools around this ditch polluted by refuse and even carrion, and in Hatay prov. (Turkey) at a strongly dung-polluted pool under a cattle shed.

In Azerbaijan, in late October, I observed Lispe orientalis during cold and bad weather, when it rained every day and usually all day long. In such conditions most flies appeared only after several hours without rain, while during the rain the only active dipteran was Lispe orientalis which hunted Diptera larvae on a wet chicken dunghill. It seems that Lispe orientalis has adapted to endure the rain and to keep on hunting because the larvae have to move up to the surface of the chicken manure and thus the prey becomes more accessible to the predator. In contrast to the related Lispe sericipalpis, Lispe orientalis prefers stagnant water. Only once near Çivlek (Turkey, Hatay prov.), I found Lispe orientalis by a rapid stream but shortly afterwards found a cattle shed in 50 metres upstream, which explained the presence of this species.

Lispe orientalis was regarded as an Oriental species, but the records listed above show that it is widespread in the South Palearctic and rather uncommon in the Oriental region where, for example, my dedicated Lispe collecting in Thailand made over several years has yielded only a single specimen of Lispe orientalis. The record from European Russia (Krasnodarsky Kray, vicinity of Sochi) is the northernmost one (43.4°N) and the first record for Europe, but I suspect that Lispe orientalis may be found in other European countries, being overlooked due to its omission from the keys for European Lispe.

## Feeding

**Figure 6.**
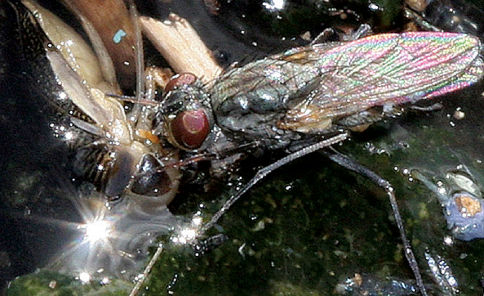
Lispe draperi Séguy, male feeding on a dead Corixidae bug (Morocco, Essaouira).

It is well known that all Lispe are predators (e.g. Werner and Pont 2006). Actually there are two poles of this behaviour. Some species are skilful hunters of active Diptera, like Dolichopodidae, Ephydridae, Muscidae or Scathophagidae. Such a hunting style is typical for the Lispe caesia species-group (note that the characteristic ventral spines on *f1* and *f2* serve to grip the prey), another example is the Oriental Lispe geniseta Stein, 1909 which usually hunts on Musca on the cattle dung in pastures. The other type is feeding on invertebrate carrion, and a typical example is the Oriental Lispe binotata Becker, 1914 which sucks dead Arthropoda from forest streams, usually ants and spiders. Species of the Lispe tentaculata group represent an intermediate type as they feed either on dead insects (Fig. 6) or on living prey, but in the later case the prey is usually an insect larvae and less often slow moving imago of a group such as Chironomidae.

## Supplementary Material

XML Treatment for Lispe alpinicola

XML Treatment for Lispe consanguinea

XML Treatment for Lispe draperi

XML Treatment for Lispe orientalis

XML Treatment for Lispe sericipalpis

XML Treatment for Lispe tentaculata
